# Drinking Water Intake Is Associated with Higher Diet Quality among French Adults

**DOI:** 10.3390/nu8110689

**Published:** 2016-10-31

**Authors:** Rozenn Gazan, Juliette Sondey, Matthieu Maillot, Isabelle Guelinckx, Anne Lluch

**Affiliations:** 1 MS-Nutrition, Faculté de médecine La Timone, AMU, Marseille 13005, France; rozenn.gazan@ms-nutrition.com (R.G.); juliette.sondey@ms-nutrition.com (J.S.); 2 Aix Marseille Univ, INSERM, INRA, NORT, Marseille 13005, France; 3 Hydration & Health Department, Danone Research, Palaiseau 91120, France; Isabelle.GUELINCKX@danone.com; 4 Danone Research, Palaiseau 91120, France; Anne.LLUCH@danone.com

**Keywords:** total water intake, drinking water intake, diet quality, nutritional index

## Abstract

This study aimed to examine the association between drinking water intake and diet quality, and to analyse the adherence of French men and women to the European Food Safety Authority 2010 Adequate Intake (EFSA AI). A representative sample of French adults (≥18) from the Individual and National Survey on Food Consumption (INCA2) was classified, by sex, into small, medium, and large drinking water consumers. Diet quality was assessed with several nutritional indices (mean adequacy ratio (MAR), mean excess ratio (MER), probability of adequate intakes (PANDiet), and solid energy density (SED)). Of the total sample, 72% of men and 46% of women were below the EFSA AI. This percentage of non-adherence decreased from the small to the large drinking water consumers (from 95% to 34% in men and from 81% to 9% in women). For both sexes, drinking water intake was associated with higher diet quality (greater MAR and PANDiet). This association remained significant independently of socio-economic status for women only. Low drinking water consumers did not compensate with other sources (beverages and food moisture) and a high drinking water intake was not a guarantee for reaching the EFSA AI, meaning that increasing consumption of water should be encouraged in France.

## 1. Introduction

Water is not only an essential nutrient for bodily and mental functions [1,2,3], it is starting to be identified as one of the key elements for chronic disease prevention [4,5,6,7,8]. In separate cohorts, lower total fluid intake [8], lower plain water intake [4], and lower 24 h urine volume [7] were all associated with increased risk for chronic kidney disease, and low water intake has also been associated with new-onset hyperglycaemia [6].

The adequate intake (AI) for total water intake (TWI) proposed by the European Food Safety Authority (EFSA) is 2.5 L/day for adult males and 2.0 L/day for adult females [9]. This dietary reference intake is less restrictive than the AI established by the US Institute of Medicine (IOM) at 3.7 L/day for men and 2.7 L/day for women [10].

Sources of TWI are fluid intake (sum of drinking water and all other beverages), food moisture, and metabolic water (derived from oxidation of macronutrients). Despite a general consensus on the major role of fluids [3], the quantitative contribution of the different sources to the TWI is lacking evidence. Based on observed fluid intake data from the National Health and Nutrition Examination Survey (NHANES) III, the IOM reported that 81% of TWI came from fluids and 19% from foods [10]. The assumption made by EFSA is that fluids contribute 70%–80% of TWI and food moisture 20%–30% [9]. A limited number of studies in Europe reported contributions of fluids to TWI ranging from 67% in Ireland up to 75% in the UK [11,12,13,14]. These ratios were means established in a population sample, and possibly masked a large variability depending on fluid intake. Documenting the contributions of water from fluids and from food moisture could be essential when translating the AI for TWI into an easy-to-understand and practical dietary guideline on fluid intake for the general population. 

In France, studies describing TWI using a representative sample of the population are scarce. Drinking water was found to be the main source of fluids in all age groups [15] with existing variations between tap water and bottled water intakes [13]. A multi-country fluid intake survey confirmed that France was characterised by a high contribution of drinking water to total fluid intake [16]. However, a study based on national population-based data of 2005–2007 estimated a TWI at 2285 mL/day for French adults aged 18–79 years old [13], suggesting that a part of the French population is at risk of inadequate intake. Considering that in 2006–2007 about 90% of children aged four to 13 years in France failed to meet the EFSA water intake recommendations [17], it seemed opportune to investigate adherence to EFSA guidelines among French adults. 

Addressing the complex delineation of the role of fluids in a healthy diet, the US-led publication the Beverage Guidance Panel suggested that the consumption of water and other beverages with no or few calories should take precedence over the consumption of beverages with more calories [18]. Further studies conducted on the US population found that an elevated consumption of drinking water—tap water and bottled water—was associated with higher nutritional quality, defined either by a healthier dietary pattern (i.e., greater consumption of vegetables, low-fat dairy products, and/or whole grains) [19], a higher food variety [20], the Healthy Eating Index (HEI) [20,21], a better micronutrient adequacy [22], or reduced energy intakes [20,21]. An elevated consumption of drinking water was also associated with higher levels of physical activity [22,23]. However, in France, there is a paucity of studies describing drinking water patterns in light of socio-demographic determinants and a complete lack of research on the association between drinking water intake and diet quality.

Based on data from a representative sample of the French adult population, the present study examined if there was an association between drinking water intake and diet quality assessed by several dietary indices. We hypothesised that the largest drinking water consumers had a better diet quality. We also estimated the adherence of men and women to the AI proposed by the EFSA and analysed the contributions of the different TWI sources. 

## 2. Materials and Methods

### 2.1. Study Population

The second Individual and National Food Consumption Survey (INCA2) was carried out by ANSES (the French Agency for Food, Environmental, and Occupational Health) between December 2005 and May 2007 among representative samples of French adults and children to collect information on habitual food and beverage consumption. The samples were obtained using a multi-stage cluster sampling technique, established by the National Institute for Statistics and Economic Studies (INSEE). The sampling frame was approved by the French National Commission for Computed Data and Individual Freedom (Commission Nationale de l’Informatique et des Libertés, CNIL). The present analyses used data from the adult sample of the INCA2 (*n* = 1918) including men (*n* = 776) and women (*n* = 1142) aged 18–79 years old. INCA2 remains the most recent version of a population-based survey available in France providing dietary intake information. A detailed survey methodology is available elsewhere [13,24].

### 2.2. Demographic, Socio-Economic and Behavioural Variables

Individual socio-economic variables were collected using a self-reported questionnaire and an interview. The following information was available: sex, age, socio-occupational status, family status, education level, income per consumption unit, food insecurity, perception of household financial situation, educational level, residency, season of protocol completion, physical activity, and smoking status. A detailed description of these variables is available elsewhere [25].

Socio-occupational status was classified into four categories: ‘low’, ‘intermediate’, ‘high’, and ‘economically inactive’. ‘High’ was assigned to executive, top-management, and professional classes; ‘intermediate’ to middle professions (office employees, technicians, and similar); and ‘low’ to manual workers and unemployed people. The fourth class, labelled as ‘economically inactive’, included retired people, students, and housewives/househusbands. 

Family status was divided into ‘couples with children’, ‘couples without children’, ‘single parent households’, and ‘single without children’. 

Education level was divided into ‘high’, ‘intermediate’, and ‘low’. ‘High’ was assigned to university education; ‘intermediate’ to high school; and ‘low’ to mid-secondary or below [26].

Income per consumption unit (ICU) was calculated as the self-reported household total net income divided by the number of consumption units in the household. The number of consumption units was calculated using the Organization for Economic Co-operation and Development (OECD) modified equivalent scale (one consumption unit for the householder, 0.5 for other household members aged 14 or over and 0.3 to each child aged less than 14 years old) [27]. For the analysis, the ICU was transformed into quintiles according to sex. 

Food insecurity was classified into ‘yes’ or ‘no’ based on the perception of the actual situation in the household about having enough food or not, and the reason for a lack of food [25]. Individuals having reported ‘getting enough, but not always the kinds of food they want to eat’, or ‘sometimes’ or ‘often not getting enough to eat’ because of ‘lack of money’ were classified as living in a household experiencing food insecurity. 

The perception of household financial situation (‘living comfortably’, ‘getting by’, ‘finding it difficult’, ‘impossible without debt’) was assessed [28] and further aggregated into two classes: ‘high’ and ‘low’. 

Residency was recorded based on eight different regions of France: Northwest; East; Ile de France; West; Centre; Centre-East; Southwest; and Southeast.

Level of physical activity was based on the International Physical Activity Questionnaire (IPAQ) score [29], which assesses physical activity, including exercise, leisure time, domestic and gardening activities, work-related and transport-related activity. 

### 2.3. Dietary Assessment

Diet was assessed using a seven-day open-ended food record. Each day of the food record was divided into three main meals (breakfast, lunch, and dinner) and three between-meals snacks. The individuals were asked to describe, as precisely as possible, all food and beverage intakes for seven consecutive days: food name, origin (home-made or industrial product), and features (low fat, low sugar, fortified, dietetic, as well as fresh, canned, or frozen). Portion sizes were expressed by weight or household measures (spoon) or estimated using a photographic booklet (SU.VI.MAX) [30]. Average daily nutritional intakes (excluding all alcoholic beverages) were evaluated matching food intakes with the 2013 French food composition database of the CIQUAL led by ANSES [31]. Daily TWI, expressed in grams per day, was estimated by assessing the amount of the nutrient “water”, from fluids and from food moisture. Energy and quantity of alcoholic beverages were estimated separately.

### 2.4. Foods and Fluids Categorization 

All the foods and fluids declared as consumed in INCA2 were categorised into nine food groups and 27 food subgroups (Supplementary Materials Table S1). In our study, the food group “drinking water” contained tap water, and non-carbonated and carbonated non-caloric bottled water. The food group “beverages” included fruit juices, hot drinks, sugar-sweetened beverages, and diet sweet beverages. “Fluids” referred to the food groups “drinking water”, “beverages”, and the “milk” subgroup. 

### 2.5. Nutritional Quality of Diet

Solid energy density (SED), mean adequacy ratio (MAR), mean excess ratio (MER), probability of adequate intakes (PANDiet) scores, and food variety were used as indicators of nutritional quality for each individual diet. The MAR, the PANDiet, and the MER are reliable indicators of the nutritional quality of diets at the population or individual level [32,33,34]. SED (kcal/100 g) was defined as the ratio of the total energy consumed from solid foods and the total weight consumed from solid foods [35,36]. A low SED diet has been associated with a good overall nutritional quality [35].

The MAR (% of adequacy) was used as an indicator of good nutritional quality and was calculated for each individual diet as the mean percentage of sex- and age-specific French Recommended Dietary Allowances (RDA) [32] for 23 key nutrients [37]. The MER (% of excess) was calculated as the mean daily percentage of the French maximum recommended values for saturated fatty acids (22.2 g), free sugars (50 g), and sodium (3153 mg), as proposed by Vieux [34]. The MAR and MER values range between 0% and 100%.

The PANDiet score was composed of adequacy probabilities for 24 nutrients grouped into two sub-scores: the adequacy sub-score (AS) and moderation sub-score (MS) [32,38]. The AS assessed the probability of adequacy for items for which the usual intake should be above a reference value, whereas the MS evaluated the probability of adequacy for several items—recently adapted and including free sugar—for which the usual intake should not exceed a reference value [38]. PANDiet scores range between 0 and 100; where 100 represents 100% of the usual intake adequacy for the 24 nutrients. 

Food variety was estimated as the number of different foods and fluids (except alcohol) declared as consumed by each individual during the seven-day food record.

### 2.6. Diet Cost

Diet cost was calculated by multiplying the quantity of each food in the diet by its mean national price. A detailed methodology has been previously described [39]. Diet cost was expressed either per day or per 2000 kcal (i.e., energy cost). Mean national prices, expressed in euros per 100 g of edible food, were previously obtained from the 2006 Kantar-World Panel database, which gives the annual food expenditures of a representative sample of 12,000 French households [40].

### 2.7. Statistical Analysis

All analyses accounted for the complex INCA2 sampling frame design [24]. Data were weighted for unequal sampling probabilities and for differential non-responses by region, agglomeration size, age, sex, occupation of the household head, size of the household and season [13,24]. All analyses were conducted separately by sex due to the sex-specific EFSA AI of TWI (>2.5 L for men and >2 L for women). Small, medium, and large consumers were identified based on the tertiles of drinking water intake (including non-consumers). Socio-demographic characteristics were described and statistically compared between tertiles of drinking water intake using the chi-squared test (for qualitative variables) and general linear models (GLM, for continuous variables). Water intakes from fluids and food moisture, expressed in g/day and in percentage of TWI, were evaluated and represented graphically. The prevalence of adherence to the AI of TWI was assessed by tertile of drinking water intake and by sex using binomial logistic regression. The distance between TWI and the AI (i.e., TWI shortfall) for individuals considered in inadequacy was assessed. Distribution of TWI and the average TWI shortfall were graphically represented by tertile of water consumption by sex, and compared two by two using GLM, with Bonferroni correction.

Food intakes, food variety, SED, MAR (%), MER (%), PANDiet, diet cost (€/day), energy cost (€/2000 kcal), and macro- and micronutrient intakes (those used in the MAR or PANDiet) were statistically compared using GLM according to tertiles of drinking water intake in observed diets. Linear trends in diet quality and food intakes were also evaluated by tertile and by sex.

A *p*-value of 5% was used as the threshold of significance. Values are survey-weighted means and adjusted for total energy intake. When specified, adjustments were made for the level of education, socio-occupational status, season, level of physical activity, smoking status, region of residence, and quintile of ICU. Based upon the weighting factors, all results are representative of the French population. Analyses were conducted using SAS version 9.4 (SAS Institute, Cary, NC, USA).

## 3. Results

### 3.1. Drinking Water Intake

**Figure 1:**
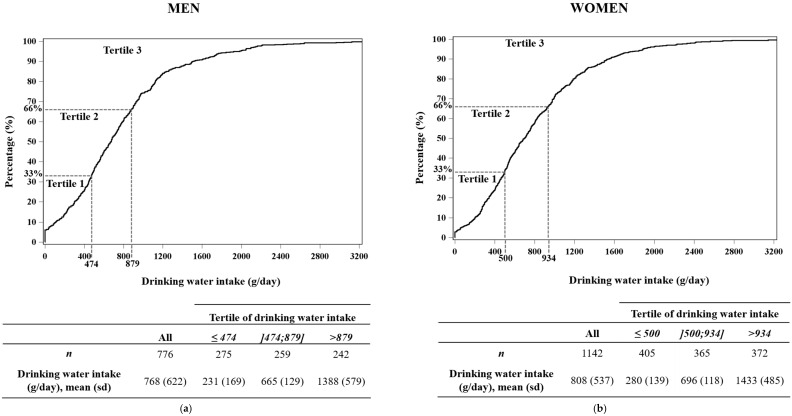
Cumulative distribution* (%) of drinking water intake and average of drinking water intake (g/day) by tertile, among men (*n* = 776) (**a**) and among women (*n* = 1142) (**b**). * The survey-weight coefficients were applied to the distribution.

Figure 1 shows the cumulative distribution of drinking water intake (g/day) by tertile and by sex. The mean intake of drinking water in the male and female sample was 768 g/day and 808 g/day, respectively. Small consumers were identified as men and women having a consumption of drinking water ≤474 g/day (including 18.3% of non-consumers) and ≤500 g/day (including 8.0% of non-consumers), respectively. Large consumers were defined as individuals consuming drinking water in an amount superior to 879 g/day among men and to 934 g/day among women.

Demographic, socio-economic, and behavioural variables by tertile of drinking water intake and by sex are presented in Table 1. Among both men and women, tertiles of drinking water intake were significantly associated with socio-occupational status and season. For men only, consumers at the highest level of drinking water intake had a significantly higher education level, higher physical activity level, higher income per consumption unit, and were more likely to be non-smokers.

### 3.2. Water Intakes from Fluids and Food Moisture

**Figure 2:**
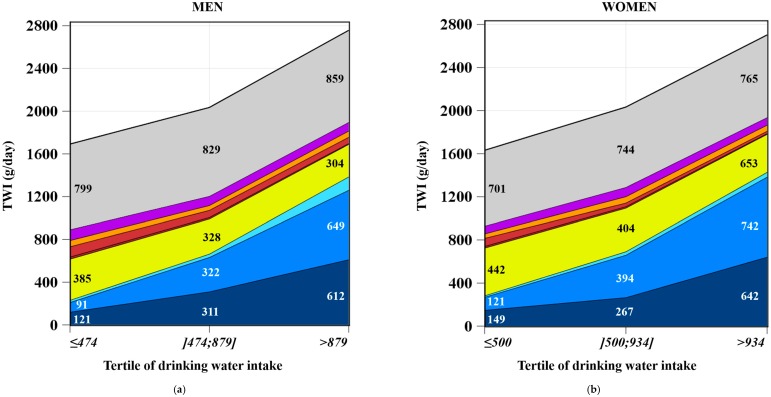

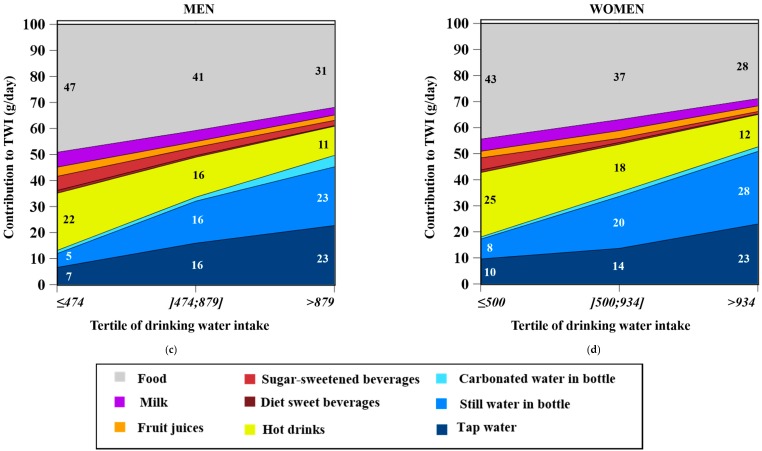
Total water intake (g/day) ^1^ by tertile of drinking water among men (*n* = 776) (**a**) and women (*n* = 1142) (**b**) (means are adjusted for energy) and the contribution (%) of fluids and food moisture to the total water intake ^2,3^ by tertile of drinking water among men (*n* = 776) (**c**) and women (*n* = 1142) (**d**). ^1^ Among men and women, *p* for trend was significant for: sugar-sweetened beverages, hot drinks, carbonated water in a bottle, still water in a bottle, and tap water; ^2^ Among men, *p* for trend was significant for: milk, fruit juices, sugar-sweetened beverages, diet sweet beverages, hot drinks, carbonated water in a bottle, still water in a bottle, and tap water; ^3^ Among women, *p* for trend was significant for: milk, sugar-sweetened beverages, diet sweet beverages, hot drinks, carbonated water in a bottle, still water in a bottle, and tap water.

Figure 2 shows the average TWI (g/day, Figure 2a,b) and the average contribution of water intake from fluids and food moisture (%, Figure 2c,d) by tertile of drinking water intake for men and women.

TWI from food moisture significantly increased from the lowest to the highest tertile (799–859 g/day among men and 701–765 g/day among women) (Figure 2a,b), while food moisture contribution to TWI decreased, both among men (from 47% to 31%, *p* for trend = 0.008) and women (from 43% to 28%, *p* for trend = 0.002) (Figure 2c,d).

Both the TWI from fluids (g/day) and fluids’ contribution to TWI (%) increased significantly from the first to the third tertile (*p* for trend < 0.0001), both among men (from 53% to 69%, i.e., 893–1897 g/day) and women (from 57% to 72%, i.e., 931–1938 g/day) (Figure 2a–d).

The contribution to TWI of all sources of drinking water (tap water, still water in a bottle, and carbonated water in a bottle) significantly increased from the lowest to the highest tertile, whereas the contribution of water from the other fluids (except fruit juices among women) significantly decreased (Figure 2c,d).

The main contributor of TWI among both men and women in the first tertile was hot drinks (with 22% for men and 25% for women) followed by tap water (with 7% for men and 10% for women), whereas in the third tertile, the main contributors were tap water (with 23% for both men and women) and still water in a bottle (with 23% for men and 28% for women). In the second tertile, contributions to TWI from hot drinks and still water in a bottle were equivalent (around 16% for men and 18%–19% for women).

### 3.3. TWI and Adherence to EFSA AI

Total water intakes by tertile of drinking water intake and by sex are presented in Table 2. The daily mean TWI was 2160.8 g/day (2.16 L) for men and 2122.3 g/day (2.12 L) for women (Table 2). 

**Figure 3:**
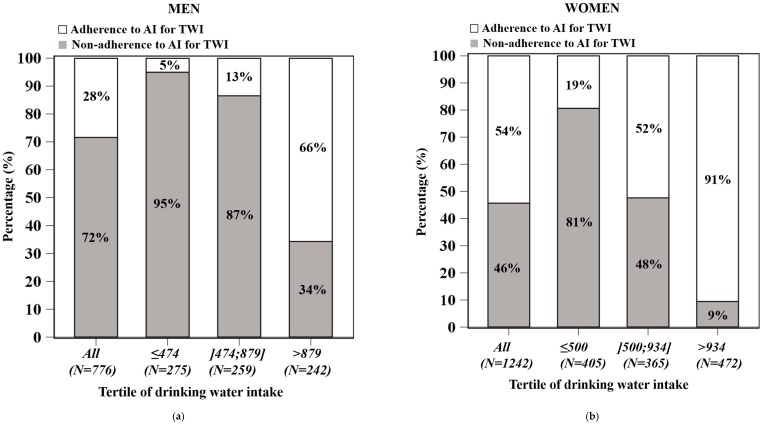

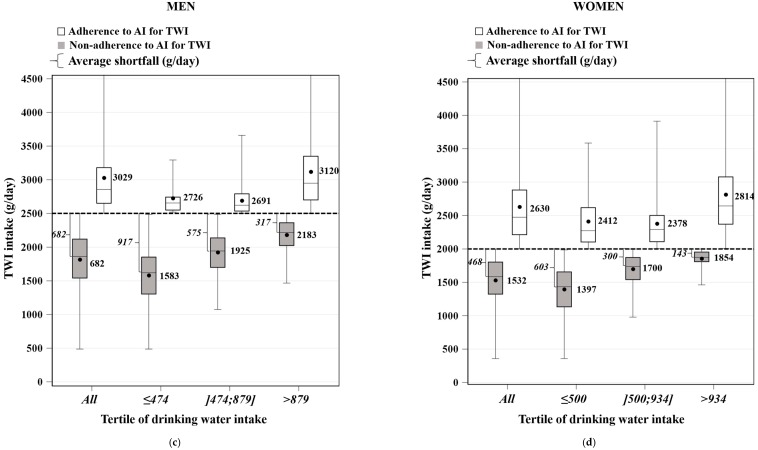
Adherence to adequate intake (AI) of total water intake (TWI) (%) by tertile of drinking water among men (*n* = 776) (**a**) and women (*n* = 1142) (**b**); survey-weighted daily average TWI in subjects meeting or failing to meet EFSA AI by tertile of drinking water among men (*n* = 776) (**c**) and women (*n* = 1142) (**d**).

Figure 3 shows the proportion of individuals (non-)adhering to the AI of TWI by tertile by drinking water intake and by sex. Seventy-two percent of men and 46% of women had a TWI below the EFSA AI (Figure 3a,c). Among both men and women, the proportion of non-adherence to AI decreased from the lowest to the highest tertile of drinking water intake (from 95% to 34%, respectively, for men, and from 81% to 9%, respectively, for women) (Figure 3a,c).

Individuals in the first and second tertile were less likely to fulfil the EFSA AI than those in the third tertile among men (*p* < 0.0001, OR = 38.8, and OR = 13.3, respectively) and women (*p* < 0.0001, OR = 49.4, and OR = 11.0 respectively) after full adjustment. No significant interaction between season and the probability of inadequacy was found (data not shown). 

The average shortfall of TWI among individuals in inadequacy significantly decreased from the lowest to the highest tertile of water consumption (917 g/day to 317 g/day for men; 603 g/day to 143 g/day for women) (*p* for trend < 0.05) (Figure 3b,d).

Among male and female individuals adhering to the AI, total TWI increased significantly from the first to the third tertile but was not different between the first and second tertile (2726 vs. 2691, *p* = 0.836 and 2412 vs. 2375, *p* = 0.627 among men and women, respectively, after adjustment) (Figure 3b,d).

### 3.4. Drinking Water Intake and Nutritional Quality of Diet

Table 2 describes the nutrient intakes and diet quality indicators of observed diets by tertile of drinking water intake and by sex. Among men and women, energy intake was significantly different between tertiles and increased (from 2290 to 2518 kcal/day, *p* for trend = 0.008 for men, and from 1779–1901 kcal/day, *p* for trend = 0.004 for women) with full adjustment. The total weight consumed also increased, steered by the strong increase of the weight of fluids from the first to the third tertile and a slight increase of the weight of solid foods (Table 2). 

For men and women, the PANDiet score and adequacy subscore, MAR (%/day), food variety, daily cost, and energy cost significantly increased with adjustment for energy intake only, from the lowest to the highest tertile (all *p* for trend < 0.05). For men only, MER (%/day) was close to a significant decrease (adjusted *p* = 0.065) and, for women only, SED decreased significantly from the first to the third tertile (*p* for trend < 0.0001) (Table 2). After full adjustments, diet cost and energy cost remained significant for men and women (*p* for trend < 0.0001), as well as PANDiet, MAR, food variety, and SED for women only (*p* for trend < 0.0001). Women with the largest consumption of drinking water intake had higher diet quality and less energy dense diets. 

For both men and women, there were no differences in macronutrient intakes, with all adjustments, except an increase of fibre from the lowest to the highest tertile (*p* for trend = 0.015 among men, and *p* for trend < 0.001 among women) and a decrease of free sugar (% energy) only for women (*p* for trend = 0.028). Only among women, intakes of sodium increased (*p* for trend < 0.001) (Table 2). Both among men and women, the level of consumption of drinking water was not strongly related to macronutrient intake. 

Vitamin and mineral intakes by tertile of drinking water and by sex are presented in Supplementary Materials Table S2. After all adjustments, significant differences were found between tertiles of drinking water. Among men, magnesium, calcium, and vitamin A intake significantly increased from the lowest to the highest tertile. Among women, linolenic fatty acid, magnesium, calcium, copper, iron, iodin, zinc, vitamin B6, folic acid, and vitamin C intake increased from the first to the third tertile. 

### 3.5. Food Intake Compared to Levels of Drinking Water Intake

The food group intakes by tertile of drinking water intake and by sex are presented in Table 3.

For both men and women, and with full adjustment, consumption of fluids was characterised by a significant increase from the lowest to the highest tertile of all types of drinking water (drinking water, tap water, still water in a bottle) (*p* for trend < 0.0001) and a decrease in other beverages (*p* for trend < 0.0001). In particular, the consumption of hot drinks significantly decreased (*p* for trend = 0.003 among men and *p* for trend = 0.005 among women) (Table 3).

For both sexes, the fruits and vegetables food group was characterised by an increase from the lowest to the highest tertile, being significant only among women (*p* for trend = 0.003 after full adjustment). This result was steered by the fresh and processed fruits subgroup (*p* for trend < 0.05 for men and *p* for trend = 0.011 for women). For men and women, after full adjustment, the consumption of fat products significantly increased from the first to the third tertile (*p* for trend = 0.001 for men and *p* for trend = 0.08 for women), steered among men by a significant increase in the vegetable fat subgroup (*p* for trend = 0.001). Among men, after full adjustment, a significant increase from the lowest to the highest tertile was found for consumption of the meat/fishes/eggs group (*p* for trend = 0.027) and a significant decrease was found for the mixed dishes and sandwiches group (*p* for trend = 0.001). Among women, sweet products consumption decreased from the first to the third tertile (*p* for trend = 0.002) and the consumption of starches and mixed dishes was significantly different between tertiles but with no linear trend (Table 3).

## 4. Discussion

This study was the first representative study of French adults investigating the associations by sex between drinking water patterns and diet quality in light of socio-economic determinants and adherence to EFSA AI for TWI. Our findings confirmed our hypothesis that an elevated drinking water intake was positively associated with diet quality, as large drinking water consumers were more likely to adhere to the EFSA AI, had diets of higher nutritional quality and, mostly among women, seemed to make healthier food choices (e.g., more fruits and vegetables and fewer sweets). Seventy-two percent of men and 46% of women in the French adult population were below the EFSA AI for TWI. In our sample, large drinking water consumers were more likely to have a high socio-occupational status and, among men only, to have a higher education level, a higher physical activity level, a higher income per consumption unit, and were more likely to be non-smokers.

It stems from our results that a higher drinking water intake was associated with higher nutritional quality of the diet, assessed by several dietary indices. In our study, the MAR and the PANDiet were positively associated with drinking water while differing in their methods, as the MAR is a simple mean percentage of sex- and age-specific French RDA [37], whereas the PANDiet is a score that takes into account different parameters, including the number of days of dietary data, the mean nutrient intake and its day-to-day variability, the nutrient reference value, and inter-individual variability [38]. Using different indices is useful to show that our observations are consistent and do not depend on a certain methodology of assessing diet quality. All relationships between tertiles of drinking water and dietary indices, SED, and energy cost, even though not all were significant, were congruent in the same direction. Full adjustment revealed that the association between drinking water intake and diet quality was particularly noticeable for women, for whom drinking water increased, as did the indicators of nutritional quality (i.e., higher MAR and PANDiet scores, and lower SED values). Results are consistent with Kim et al., who found a positive association, among both Korean men and women, between drinking water intake and the MAR [41]. When not using dietary indices, other relationships between drinking water intake and nutrients have been found in the literature that we could consider as good indicators of nutritional quality. In the US, Yang and Chun found a positive association between TWI, drinking water, and moisture in foods with dietary and serum minerals, vitamins, and carotenoids [22]. Those nutrients positively associated with TWI can be compared to identical components of the MAR, and are in line with our results. In a recent national study of US adults, an increase in the proportion of daily drinking water in TWI was found to be associated with a decreased daily intake of total energy, energy from sugar-sweetened beverages, discretionary foods, and total fat, saturated fat, sugar, sodium, and cholesterol [21]. Most of those nutrients, the intake of which is negatively associated with drinking water, are similar to the components of the MER, and are also slightly decreased in our results among men. Higher intakes of sodium found in women could possibly explain why the MER did not significantly decrease. In previous studies, a positive association was found between drinking water and sodium intakes [4,41], similar to our results among women, although the cause-consequence relationship is unknown.

In terms of fluid patterns, our results indicate that large drinking water consumers seemed to favour a higher intake of only water and not necessarily all other fluids. A similar observation was made by Illescas-Zarate et al. in her study among Mexican adults, in which a negative association was found between drinking water intake and sugar-sweetened beverages [42]. 

In terms of food choices, large drinking water consumers seemed to favour diets rich in moisture-abundant foods, especially fresh fruits. This finding is in line with the literature reporting the opposite observation, i.e., low levels of fruit and vegetable intake among low drinking water consumers [23,41]. Similarly, a negative association between drinking water intake and beverage moisture, but a positive association with food moisture, was found among US adults [20,22]. Kant et al. suggested some substitution effect of drinking water intake on other fluid consumption and possibly higher fruit and vegetable intake [20]. According to Hedrick et al., consumption of water, unsweetened tea/coffee, low-fat milk, artificially sweetened beverages, and fruit/vegetable juice is closely aligned with a “prudent” dietary pattern (usually including vegetables, fruits, legumes, whole grains, fish, and poultry); conversely, the consumption of high-fat milk, alcohol, and sugar-sweetened beverages is strongly associated with a “Western” dietary pattern (usually including red meat, processed meat, refined grains, sweets and dessert, French fries, and high-fat dairy products) [43]. This last point is particularly illustrated in our results with the decrease of mixed dishes and sandwiches among men and of sweet products among women from the first to third tertile of drinking water intake. Large drinking water consumers tended to consume more moisture-abundant foods, also rich in nutrients and vitamins, implying that people fulfilling their water intake requirements have higher quality diets and make food choices more likely to fulfil their nutritional requirements.

Identifying characteristics of large drinking water consumers can be useful to identify individuals more likely to have healthier dietary behaviours. In the literature, associations have been found between drinking water intake and some socioeconomic determinants, such as sex [42,44], age [22,23,41,45], education level [22,23], income [23,45], level of physical activity [22,23,41], and smoking status [11,23,41]. The novelty of our study is that drinking water patterns were explored in light of several factors combined (socio-demographics, lifestyle determinants, and overall dietary intake). Moreover, a major strength of our study is the investigation of socioeconomic determinants of drinking water intake by sex in order to identify gender specificities, which has only been done before, to our knowledge, by Kim et al. [41]. In our study, both among men and women, drinking water intake was positively associated with socioeconomic position. However, female drinking water intake appears to be less influenced by socioeconomic factors, considering the numerous additional associations for men, notably with education, physical activity, income, and smoking status. Thus, among men, drinking water intake may be considered as a reliable indicator of socioeconomic differences. This male specificity has not been noticed by Kim et al. among Korean adults [41]. Among men, the association between drinking water intake and diet quality was explained by their socio-economic status, as men in higher socio-economic strata had a higher nutritional quality and higher drinking water intake. However, among women, the association between drinking water intake and a healthy dietary pattern was independent of the social level. Gender contrasts in food choices are influenced by views on food and health, the ethical dimensions of food production and food selection, nutritional attitudes and choices, dietary change, food work, and body image [46]. One hypothesis could be that, regarding beverages, women appear to be more ‘health-conscious’ [47] than males, who consume more alcohol [14,48,49] and sweet beverages regularly [14,49]. More research is needed to fully apprehend divergent associations found in the literature and to verify if women and men follow a distinct drinking water pattern elsewhere than in France.

In the current study, daily means of TWI were consistent with results from previous European studies [11,14,50]. However, a remarkable observation was made in that the sex difference in TWI was less pronounced in France than in some other countries: estimated TWI was slightly lower for men and slightly higher for women than those of Irish [11], British [14], and German [50] adults (2.16 L vs. 2.52 L, 2.53 L, and 2.48 L for men, and 2.12 L vs. 2.09 L, 2.03 L, and 2.05 L for women, respectively). This is, on one hand, in contrast to a tendency reported in the literature with men systematically having a higher TWI than women [4,11,14,20,22,45,50] while, on the other hand, fluid surveys in 13 countries showed few significant differences in fluid intake between both sexes. The latter suggests that if there is a gender difference in TWI, the difference could be due to a difference in water from food moisture, a result that was also observed in our study, but needs to be confirmed further.

Several strengths and limitations of this study should be acknowledged. The INCA2 data were collected in 2006–2007 and the observations may already be obsolete. However, INCA2 is still the most recent version of a population-based survey available in France and remains the standard source of reliable information about dietary intakes. Furthermore, between the first version of the national survey (INCA1) and the second (INCA2), the food group ‘waters’ increased from six items to 50 items, increasing the robustness of data from this food group, which is of particular importance for the present study [51]. A potential additional limitation could come from numerous low drinking water consumers interviewed during the winter. However, no interaction effect was found between the season and drinking consumption patterns on the level of adequacy (data not shown). Another limitation, inherent to any dietary survey, is the fact that the nutritional intake data is self-reported. Especially collecting data to evaluate TWI is not without limitations: accurate recording of drinking water and other beverages, as well as estimating water from food moisture, might be prone to bias [52]. A final point of discussion is the exclusion of alcohol. Since several publications report a major contribution of alcoholic beverages to water intake [11,14,22,41,45], excluding these beverages could lead to an overestimation of the proportion of French adults below the EFSA AI of TWI. However, this could also be interpreted as a limitation in our case, since having an adequate intake of TWI should be achieved mainly with drinking water. 

## 5. Conclusions

This is the first description of total water intakes among small, medium, and large drinking water consumers considering socio-demographic determinants and diet quality among men and women in France. It shows that large drinking water consumers have healthier fluid intake and nutritional patterns, independent of the social level among women. In the future, more research should be performed to demonstrate the role of a high water intake, as part of a high quality diet, in the prevention of chronic disease. In the meantime, these results could already imply that the guidelines for disease prevention should not only mention a high quality diet, but also a high intake of water. Nevertheless, inadequacy of TWI remains prevalent at all levels of drinking water intake for both sexes. Advice regarding the importance of drinking water intake is still necessary to help individuals to reach adequate water intake.

## Figures and Tables

**Table 1:** Demographic, socio-economic, and behavioural variables by tertile of drinking water intake (g/day) and by sex ^1^.

	Men					Women				
	Tertile of Drinking Water Intake					Tertile of Drinking Water Intake				
	All	≤474 mL/Day	474–879 mL/Day	>879 mL/Day	*p*	All	≤500 mL/Day	500–932 mL/Day	>934 mL/Day	*p*
*n*	776	275	259	242			1142	405	365	
	Mean ± SD					Mean ± SD				
Age (years)	49.0 ± 17.8	50.5 ± 16.9	48.7 ± 19.1	47.9 ± 17.2	0.333	45.2 ± 15.0	45.4 ± 15.1	45.1 ± 15.4	45.0 ± 14.6	0.977
	(%)					(%)				
Socio-occupational status					0.001					0.009
Low	20.4	19.1	18.1	23.9		10.1	6.9	10.1	13.4	
Intermediate	26.2	25.2	21.2	32.0		40.4	41.7	38.4	41.0	
High	10.7	8.6	11.1	12.2		7.3	5.7	5.5	10.6	
Economically inactive	42.7	47.1	49.6	31.9		42.2	45.7	46.1	34.9	
Familial status					0.182					0.204
Couple with children	26.5	25.7	22.7	30.8		33.0	33.3	31.5	34.2	
Couple without child	47.9	52.2	47.2	44.4		34.4	29.6	35.8	37.8	
Single parent household	6.3	4.9	8.8	5.2		7.0	9.1	5.9	6.0	
Single without children	19.3	17.2	21.3	19.5		25.4	27.9	26.4	22.1	
No answer	0.0	0.0	0.0	0.0		0.1	0.0	0.4	0.0	
Quintile of ICU ^†^					0.009					0.154
1	20.2	25.2	18.2	17.3		20.6	23.5	20.9	17.5	
2	19.3	21.3	22.3	14.3		19.9	19.1	19.2	21.2	
3	23.1	14.9	27.3	26.9		19.6	21.5	18.9	18.6	
4	19.6	19.2	17.8	21.8		23.2	20.7	24.9	24.0	
5	17.8	19.4	14.3	19.8		16.7	15.1	16.1	18.7	
Food insecurity					0.339					0.303
Yes	9.9	9.3	11.9	8.3		11.9	14.0	10.2	11.5	
No	85.9	84.2	83.7	89.6		83.2	81.7	86.0	81.9	
No answer	4.3	6.4	4.3	2.1		4.9	4.4	3.8	6.6	
Perception of household financial situation					0.242					0.174
High	6.4	8.7	5.3	5.3		94.7	92.8	94.8	96.6	
Low	93.0	90.5	93.9	94.6		5.0	7.0	4.7	3.4	
No answer	0.5	0.7	0.8	0.1		0.3	0.2	0.6	0.0	
Level of education					0.010					0.599
Low	15.4	21.0	17.0	8.4		20.1	22.1	20.2	18.1	
Intermediate	55.5	53.4	53.8	59.2		48.6	49.1	49.5	47.2	
High	29.0	25.7	29	32.2		31.2	28.8	30.0	34.7	
No answer	0.1	0.0	0.2	0.2		0.1	0.0	0.3	0.0	
Region of residence					0.128					0.008
Northwest	13.8	12.8	13.3	15.2		15	11.2	19	15	
East	8.9	10.0	11.2	5.6		9.7	8.5	8.7	11.8	
Ile De France	17.7	19.7	14.7	18.8		17.3	22.5	15.0	14.3	
West	14.1	15.2	13.8	13.3		14.4	12.5	18.1	12.6	
Centre	10.4	10.4	13.6	7.4		9.6	10.3	9.5	9.1	
Centre-east	11.6	10.1	7.9	16.5		12.6	14.8	12.2	10.8	
South-west	11.2	10.4	12.8	10.3		10.0	9.2	6.2	14.4	
South-east	12.4	11.5	12.6	13.0		11.5	11.1	11.4	12.0	
Season of protocol completion					0.004					<0.001
Winter	23.8	27.4	25.0	19.0		26.9	33.1	25.3	22.5	
Spring	26.4	22.4	25.4	31.4		23.5	23.6	22.1	24.7	
Summer	24.0	21.2	20.3	30.3		26.9	16.8	28.9	35.0	
Autumn	25.9	29.0	29.3	19.4		22.6	26.5	23.7	17.8	
Level of physical activity (IPAQ score)					0.017					0.808
Low	20.7	24.6	20.6	17.0		24.0	23.3	24.3	24.5	
Middle	29.6	31.8	33.9	23.2		32.4	34.2	33.4	29.7	
High	48.6	43.3	45.3	56.9		42.4	41.6	41.1	44.5	
No answer	1.1	0.2	0.2	2.8		1.1	0.9	1.2	1.2	
Smoker					0.004					0.164
Smoker	28.1	36.5	22.3	25.7		23.3	26.9	19.9	23.0	
Not smoker	71.9	63.5	77.7	74.3		76.7	73.1	80.1	77.0	

Abbreviations: ICU, income per consumption unit; IPAQ, International Physical Activity Questionnaire. ^1^ Values are survey-weighted means with standard deviations; ^†^ quintiles of ICU were ≤700; ]700;976]; ]976;1367]; ]1367;1867]; >1867 and ≤580; ]580;915]; ]915;1306]; ]1306;1867]; among men and women, respectively.

**Table 2:** Observed nutrient intakes and diet quality indicators by tertile of drinking water intake (g/day) and by sex (means are adjusted for energy) ^1^.

	Men						
		Tertile of Drinking Water Intake					
	All	≤474 mL/Day	474–879 mL/Day	>879 mL/Day			
Variables	Mean ± SD	Mean ± SD	Mean ± SD	Mean ± SD	*p* ^†^	*p* ^‡^	*p* for Trend ^§^
Energy (kcal/day)	2403.8 ± 592.0	2289.7 ± 529.3	2400.1 ± 551.3	2518.0 ± 671.2	<0.001	0.003	0.001
Total water (TWI ^2^, g/day)	2160.8 ± 28.8	1691.6 ± 25.2	2034.1 ± 19.9	2756.7 ± 41.2	<0.001	<0.001	<0.001
Water from foods (g/day)	829.1 ± 16.9	798.9 ± 15.7	829.2 ± 18.0	859.3 ± 17.2	0.041	0.032	0.009
Water from fluids (g/day)	1331.7 ± 27.3	892.7 ± 24.1	1204.9 ± 17.9	1897.3 ± 40.0	<0.001	<0.001	<0.001
Total weight (g/day)	2668.3 ± 29.1	2199.9 ± 25.3	2540.4 ± 20.4	3264.7 ± 41.7	<0.001	<.0001	<0.001
Weight of solid foods (g/day)	1304.6 ± 18.1	1270.4 ± 17.4	1306.0 ± 18.8	1337.3 ± 18.3	0.036	0.022	0.006
Weight of fluids (g/day)	1363.8 ± 28.3	929.5 ± 25.6	1234.3 ± 18.6	1927.5 ± 40.6	<0.001	<0.001	<0.001
Food variety	56.1 ± 1.1	53.2 ± 1.0	57.6 ± 1.2	57.5 ± 1.1	0.003	0.121	
Energy from alcohol	180.4 ± 194.4	216.2 ± 211.9	154 ± 171.4	171.5 ± 191.1	0.008	0.007	0.030
Alcoholic drinks	255.4 ± 21.2	294.7 ± 20.9	222.8 ± 22.4	248.7 ± 20.4	0.053	0.077	
SED (kcal/100 g)	179.9 ± 2.1	183.0 ± 2.2	180.0 ± 2.3	176.8 ± 2.0	0.119	0.084	
PANDiet	62.5 ± 0.5	61.4 ± 0.4	62.6 ± 0.5	63.7 ± 0.5	0.001	0.084	
Adequacy subscore	69.6 ± 0.7	68.1 ± 0.7	69.7 ± 0.7	71.0 ± 0.6	0.005	0.274	
Moderate subscore	55.5 ± 0.7	54.7 ± 0.7	55.4 ± 0.7	56.3 ± 0.7	0.235	0.299	
MAR (% adequacy)	83.3 ± 0.4	82.3 ± 0.5	83.4 ± 0.4	84.2 ± 0.4	0.006	0.093	
MER (% excess)	44.4 ± 1.2	47.2 ± 1.2	43.9 ± 1.2	42.1 ± 1.2	0.004	0.065	
Cost (€/day)	7.3 ± 0.1	7.0 ± 0.1	7.3 ± 0.1	7.7 ± 0.1	<0.001	<0.001	<0.001
Cost (€/2000 kcal)	6.2 ± 0.2	5.8 ± 0.2	6.1 ± 0.2	6.5 ± 0.2	<0.001	0.084	
Proteins (% energy)	16.9 ± 2.9	17.0 ± 3.3	17.0 ± 2.8	16.6 ± 2.7	0.326	0.369	
Carbohydrates (% energy)	43.0 ± 6.9	43.0 ± 7.2	42.6 ± 6.6	43.4 ± 7.0	0.459	0.751	
Total fat (% energy)	37.9 ± 6.2	37.8 ± 6.0	38.2 ± 5.9	37.7 ± 6.7	0.681	0.885	
Saturated fat (% energy)	14.6 ± 3.3	14.9 ± 3.1	14.7 ± 3.2	14.3 ± 3.6	0.141	0.306	
Free sugar (% energy)	9.0 ± 5.6	9.4 ± 5.9	8.7 ± 5.3	8.9 ± 5.5	0.372	0.243	
Fiber (g) ^†^	20.6 ± 0.3	20.1 ± 0.3	20.5 ± 0.3	21.1 ± 0.4	0.099	0.038	0.015
Saturated fat (g/day)	39.3 ± 0.6	39.9 ± 0.5	39.4 ± 0.5	38.5 ± 0.7	0.229	0.421	
Free sugar (g/day)	56.0 ± 2.2	59.7 ± 2.3	54.6 ± 2.3	53.7 ± 2.1	0.119	0.105	
Sodium (mg/day)	3664.1 ± 49.9	3652.5 ± 45.4	3684.0 ± 58.4	3655.8 ± 45.7	0.895	0.769	
	**Women**						
		**Tertile of Drinking Water Intake**					
	**All**	**≤500 mL/Day**	**500–934 mL/Day**	**>934 mL/Day**			
**Variables**	**Mean ± SD**	**Mean ± SD**	**Mean ± SD**	**Mean ± SD**	***p*** **^†^**	***p*** **^‡^**	***p*** **for Trend ^§^**
Energy (kcal/day)	1866.1 ± 427.1	1779.4 ± 421.9	1910.9 ± 407.7	1908.6 ± 440.5	0.001	0.003	0.004
Total water (TWI ^2^, g/day)	2122.3 ± 34.2	1631.4 ±33.5	2033.0± 34.8	2702.6 ± 34.4	<0.001	<0.001	<0.001
Water from foods (g/day)	736.3 ± 13.7	700.9 ± 14.9	743.5 ± 13.0	764.5 ± 13.1	0.004	0.006	0.002
Water from fluids (g/day)	1386.1 ± 30.2	930.6 ± 25.2	1289.5 ± 35.5	1938.1 ± 29.9	<0.001	<0.001	<0.001
Total weight (g/day)	2512.2 ± 34.7	2021.0 ± 33.8	2422.3 ± 35.6	3093.4 ± 34.8	<0.001	<0.001	<0.001
Weight of solid foods (g/day)	1100.2 ± 14.6	1063.0 ± 15.8	1105.9 ± 14.0	1131.7 ± 14.0	0.004	0.005	0.001
Weight of fluids (g/day)	1412.1 ± 30.7	958.0 ± 25.9	1316.4 ± 36.3	1961.8 ± 29.9	<0.001	<0.001	<0.001
Food variety	59.8 ± 0.8	55.5 ± 0.6	62.9 ± 1.0	61.1 ± 0.8	<0.001	<0.001	<0.001
Energy from alcohol	49.5 ± 70.5	45.1 ± 65.4	53.4± 67.1	50.2 ± 78.5	0.397	0.257	
Alcoholic drinks	63.2 ± 6.0	57.9 ± 5.7	66.2 ± 5.8	65.4 ± 6.5	0.547	0.438	
SED (kcal/100 g)	165.5 ± 2.0	171.7 ± 2.5	164.3 ± 1.9	160.5 ± 1.7	0.001	<0.001	<0.001
PANDiet	62.3 ± 0.5	60.6 ± 0.5	62.6 ± 0.4	63.6 ± 0.5	<0.001	0.001	<0.001
Adequacy subscore	64.3 ± 0.6	60.7 ± 0.6	65.8 ± 0.5	66.5 ± 0.6	<0.001	<0.001	<0.001
Moderate subscore	60.2 ± 0.7	60.5 ± 0.6	59.3 ± 0.7	60.7±0.7	0.295	0.188	
MAR (% adequacy)	79.1 ± 0.4	76.4 ± 0.5	79.9 ± 0.4	80.8 ± 0.4	<0.001	<0.001	<0.001
MER (% excess)	21.7 ± 0.9	23.3 ± 1.1	21.7 ± 1.0	20.2 ± 0.7	0.089	0.199	
Cost (€/day)	6.2 ± 0.2	5.8 ± 0.2	6.3 ± 0.2	6.5 ± 0.2	<0.001	<0.001	<0.001
Cost (€/2000 kcal)	6.8 ± 0.1	6.3 ± 0.1	6.9 ±0.1	7.2 ± 0.1	<0.001	<0.001	<0.001
Proteins (% energy)	16.2 ± 2.7	16.2 ± 2.8	16.1 ± 2.7	16.3 ± 2.7	0.608	0.810	
Carbohydrates (% energy)	42.5 ± 5.7	42.8 ± 6.1	42.3 ± 5.9	42.4 ± 5.2	0.507	0.427	
Total fat (% energy)	38.8 ± 5.3	38.6 ± 5.3	39.2 ± 5.5	38.8 ± 5.1	0.552	0.388	
Saturated fat (% energy)	14.6 ± 2.8	14.6 ± 2.9	14.8 ± 2.7	14.6 ± 2.9	0.592	0.508	
Free sugar (% energy)	9.9 ± 4.8	10.2 ± 5.7	10.2 ± 4.4	9.4 ± 4.0	0.066	0.028	0.028
Fibre (g) ^†^	16.9 ± 0.3	16.3 ± 0.2	16.7 ± 0.3	17.5 ± 0.2	0.003	<0.001	<0.001
Saturated fat (g/day)	30.4 ± 0.4	30.3 ± 0.3	30.6 ± 0.4	30.3 ± 0.4	0.769	0.662	
Free sugar (g/day)	47.4 ± 1.5	49.8 ± 1.7	48.1 ± 1.5	44.2 ± 1.2	0.015	0.006	0.003
Sodium (mg/day)	2687.1 ± 40.6	2577.2 ± 30.8	2712.3 ± 54.8	2771.9 ± 36.4	<0.001	<0.001	<0.001

Abbreviations: TWI, total water intake; SED, solid energy density; PANDiet, probability of adequate intakes; MAR, mean adequacy ratio; MER, mean excess ratio. ^1^ Values are survey-weighted means with standard deviations; ^2^ TWI, total water intake; ^†^ adjustment for energy intake (except for energy and variables expressed in %energy); ^‡^ adjustment for energy intake (except for energy and variables expressed in %energy), level of education, socio-occupational group, season, level of physical activity, smoker status, region, and quintile of income per consumption unit (ICU); ^§^ calculated only for significant differences.

**Table 3:** Food intakes by food groups and subgroups by tertile of drinking water (g/day) and by sex (means are adjusted for energy) ^1^.

	Men										
			Tertile of Drinking Water Intake								
	All		≤474 mL/Day		474–879 mL/Day		>879 mL/Day				
Food Groups and Subgroups (g/Day)	Mean ± SD		Mean ± SD		Mean ± SD		Mean ± SD		*p* ^†^	*p* ^‡^	*p* for Trend ^§^
Fruits and vegetables	368.1 ± 19.1		336.6 ± 16.3		370.4 ± 21.3		397.4 ± 19.7		0.069	0.076	
Vegetables, soup and crudités	210.0 ± 11.6		201.2 ± 12.1		215.1 ± 11.8		213.6 ± 10.8		0.602	0.449	
Fresh and processed fruits	155.9 ± 11.1		132.4 ± 7.6		153.3 ± 12.3		181.9 ± 13.4		0.008 *	0.048 *	0.015
Nuts	2.3 ± 0.4		3.0 ± 0.5		2.0 ± 0.4		1.9 ± 0.3		0.139	0.209	
Starches	306.3 ± 7.5		303.1 ± 8.1		304.6 ± 6.2		311.3 ± 8.0		0.742	0.355	
Refined starches	212.0 ± 6.3		206.0 ± 7.5		210.7 ± 5.9		219.4 ± 5.4		0.316	0.093	
Unrefined starches	89.8 ± 4.0		91.3 ±3.7		89.6±3.4		88.5 ± 4.9		0.907	0.869	
Cereals for breakfast	4.5 ± 1.3		5.8 ± 1.8		4.4 ± 1.1		3.4 ± 1.0		0.504	0.200	
Meats/fishes/eggs	190.9±4.7		181.1 ± 4.6		194.5 ± 4.4		197.3 ± 5.1		0.026 *	0.034 *	0.027
Eggs	16.2 ± 1.2		15.2 ± 1.1		17.1 ± 1.3		16.2 ± 1.3		0.530	0.503	
Fishes	30.2 ± 2.1		27.6 ± 2.1		31.9 ± 2.1		31.2 ± 2.1		0.271	0.514	
Meat	144.6 ± 4.6		138.3 ± 4.6		145.5 ± 4.1		149.9 ± 5.2		0.270	0.253	
Mixed dishes and sandwiches	153.6 ± 7.6		175.1 ± 7.3		148.3 ± 8.6		137.4 ± 6.9		<0.001 *	0.001 *	0.001
Ready-made dishes and stocks	90.9 ± 5.6		101.4 ± 5.1		90.6 ± 5.5		80.7 ± 6.2		0.018 *	0.063	
Sandwiches and savoury puff pastries	62.7 ± 5.3		73.7 ± 6.1		57.6 ± 5.0		56.7 ± 4.8		0.018 *	0.002 *	0.001
Dairy products	214.1 ± 14.3		219.3 ± 20.9		211.1 ± 11.9		211.7 ± 10.0		0.924	0.603	
Milk	101.9 ± 13.1		114.7 ± 20.2		98.0 ± 10.5		93.1 ± 8.5		0.563	0.247	
Fresh dairy products	70.9 ± 5.8		61.4 ± 5.1		71.2 ± 5.1		80.1 ± 7.4		0.075	0.167	
Cheese	41.2 ± 2.8		43.2 ± 2.8		42.0 ± 2.7		38.5 ± 2.8		0.332	0.502	
Sweet products	123.6 ± 4.7		126.4 ± 4.6		125.9 ± 4.9		118.5 ± 4.6		0.367	0.601	
Dairy dessert	18.6 ± 2.2		20.6 ± 2.2		17.5 ± 2.2		17.8 ± 2.2		0.550	0.532	
Cakes, tarts, sweet pastries	67.5 ± 3.8		67.8 ± 4.2		70.0 ± 4.1		64.6 ± 3.1		0.585	0.564	
Biscuits and sweets	37.5 ± 2.4		38.0 ± 2.1		38.4 ± 3.1		36.0 ± 2.0		0.791	0.774	
Drinking water	761.5 ± 18.5		232.4 ± 11.1		664.8 ± 8.5		1387.3 ± 35.8		<0.001 *	<0.001 *	<0.001
Tap water	348.2 ± 24.2		120.9 ± 11.3		311.2 ± 22.2		612.6 ± 38.9		<0.001 *	<0.001 *	<0.001
Still water in a bottle	354.1 ± 28.1		90.7 ± 10.9		322.3 ± 24.1		649.2±49.3		<0.001 *	<0.001 *	<0.001
Carbonated water in a bottle	59.2 ± 14.0		20.8 ± 4.7		31.3 ± 5.5		125.5±31.8		0.005 *	0.025 *	0.007
Beverages	500.3 ± 21.1		582.4 ± 21.9		471.5 ± 21.3		447.0 ± 20.2		<0.001 *	<0.001 *	<0.001
Hot drinks (Tea. Coffee)	343.6 ± 18.5		389.8 ± 20.4		332.4 ± 17.4		308.7 ± 17.7		0.011 *	0.010 *	0.003
Diet sweet beverages	14.4 ± 4.8		20.3 ± 5.8		14.8 ± 5.7		8.1 ± 3.0		0.086	0.351	
Sugar-sweetened beverages	82.2 ± 12.4		106.8 ± 14.7		74.0 ± 10.9		65.9 ± 11.5		0.035 *	0.055	
Fruit juices	60.1 ± 6.7		65.6 ± 8.4		50.3 ± 4.9		64.4 ± 6.9		0.092	0.064	
Fat products	47.0 ± 1.9		42.5 ± 1.5		47.8 ± 2.1		50.6 ± 2.0		0.003 *	0.003 *	0.001
Animal fat	14.7 ± 0.9		14.7 ± 0.8		14.6 ± 1.2		14.7 ± 0.8		0.993	0.844	
Vegetable fat	23.2 ± 1.4		19.7 ± 1.3		23.6 ± 1.6		26.3 ± 1.2		0.001 *	0.001 *	<0.001
Spices and sauces	9.1 ± 0.9		8.2 ± 0.7		9.6 ± 0.7		9.6 ± 1.3		0.183	0.286	
	**Women**										
			**Tertile of Drinking Water Intake**								
	**All**		**≤500 mL/Day**		**500–934 mL/Day**		**>934 mL/Day**				
**Food Groups and Subgroups (g/Day)**	**Mean**	**SD**	**Mean**	**SD**	**Mean**	**SD**	**Mean**	**SD**	***p*** **^†^**	***p*** **^‡^**	***p*** **for Trend ^§^**
Fruits and vegetables	374.7	13.4	345.8	16.9	378.7	12.5	399.5	10.7	0.047	0.008	0.003
Vegetables, soup and crudités	213.7	8.6	203.0	10.3	216.4	7.9	221.6	7.7	0.333	0.105	
Fresh and processed fruits	159.3	8.3	141.2	8.5	160.5	7.5	176.0	8.8	0.025	0.037	0.011
Nuts	1.8	0.3	1.6	0.2	1.8	0.3	1.9	0.3	0.777	0.712	
Starches	202.4	4.8	207.7	5.4	192.8	4.1	206.7	5.1	0.019	0.005	0.551
Refined starches	126.8	3.5	130.4	3.2	120.7	3.4	129.1	3.9	0.084	0.084	
Unrefined starches	70.3	3.5	73.5	3.7	66.6	2.8	70.9	3.9	0.342	0.223	
Cereals for breakfast	5.4	1.0	3.8	0.8	5.5	1.0	6.7	1.1	0.056	0.299	
Meats/fishes/eggs	138.1	3.0	134.7	2.7	137.8	3.3	141.8	3.0	0.165	0.579	
Eggs	14.4	1.3	15.0	1.6	12.9	1.0	15.4	1.4	0.285	0.194	
Fishes	29.8	1.6	27.9	1.4	31.3	1.9	30.3	1.5	0.229	0.345	
Meat	93.9	2.7	91.9	2.5	93.7	2.9	96.2	2.6	0.495	0.793	
Mixed dishes and sandwiches	106.5	4.5	107.7	5.8	113.1	4.0	98.8	3.8	0.035	0.003	0.214
Ready-made dishes and stocks	64.8	3.3	61.7	2.9	70.8	3.7	61.8	3.2	0.122	0.043	
Sandwiches and savoury puff pastries	41.7	2.9	46.0	4.2	42.3	2.4	36.9	2.1	0.159	0.008	0.023
Dairy products	199.4	9.6	185.5	8.8	213.1	11.2	199.5	8.7	0.182	0.353	
Milk	86.3	7.6	82.0	6.3	97.3	8.3	79.5	8.2	0.217	0.240	
Fresh dairy products	86.6	4.6	78.5	4.1	89.2	5.0	92.0	4.6	0.080	0.603	
Cheese	26.5	1.4	25.0	1.3	26.7	1.3	27.9	1.6	0.309	0.114	
Sweet products	111.9	4.2	114.9	3.5	116.1	5.7	104.7	3.5	0.047	0.004	0.002
Dairy dessert	18.1	2.3	16.9	1.4	19.7	3.2	17.8	2.4	0.734	0.711	
Cakes, tarts, sweet pastries	61.1	3.4	62.8	3.6	65.2	3.9	55.2	2.6	0.057	0.013	
Biscuits and sweets	32.7	1.8	35.2	2.0	31.2	1.6	31.7	1.9	0.238	0.419	
Drinking water	802.5	14.9	284.0	8.3	693.2	8.1	1430.3	28.2	<0.001	<0.001	<0.001
Tap water	352.8	23.6	148.8	8.3	266.7	18.0	642.9	44.6	<0.001	<0.001	<0.001
Still water in a bottle	419.2	21.6	121.2	8.5	394.1	20.1	742.4	36.3	<0.001	<0.001	<0.001
Carbonated water in a bottle	30.5	5.2	14.0	3.6	32.3	5.2	45.1	6.7	<0.001	<0.001	<0.001
Beverages	523.3	29.5	592.0	27.5	526.0	35.7	451.9	25.4	<0.001	<0.001	<0.001
Hot drinks (Tea. Coffee)	404.1	27.8	447.0	23.8	408.4	34.6	356.8	25.1	0.011	0.018	0.005
Diet sweet beverages	12.2	3.5	16.2	4.9	13.7	3.9	6.8	1.6	0.016	0.014	0.100
Sugar-sweetened beverages	48.4	10.4	82.9	17.6	37.5	9.5	24.8	4.2	0.006	0.007	0.002
Fruit juices	58.6	6.4	45.9	8.7	66.3	5.2	63.5	5.2	0.069	0.049	0.150
Fat products	45.1	1.2	43.0	0.9	45.1	1.1	47.1	1.4	0.033	0.029	0.008
Animal fat	13.3	0.8	13.5	0.7	12.9	0.7	13.6	0.8	0.786	0.489	
Vegetable fat	23.4	0.9	22.2	0.8	23.6	0.8	24.5	1.1	0.117	0.189	
Spices and sauces	8.3	0.7	7.3	0.6	8.6	0.5	9.0	0.9	0.198	0.300	

^1^ Values are survey-weighted means with standard deviations; ^†^ adjustment for energy intake; ^‡^ adjustment for energy intake, level of education, socio-occupational group, season, level of physical activity, smoker status, region, and quintile of income per consumption unit (ICU); ^§^ calculated only for significant differences.

## Supplementary Materials

The following are available online at http://www.mdpi.com/2072-6643/8/11/689/s1, Table S1: Nine food groups and 27 food subgroups, Table S2: Vitamin and mineral intakes by sex and tertile.
