# Supplementary material for: Drinking Water Intake Is Associated with Higher Diet Quality among French Adults
Source: Nutrients. 2016 Oct 31;8(11):689. doi: 10.3390/nu8110689 (PMC5133077; doi:10.3390/nu8110689)
Supplement: Supplementary file 1 [file nutrients-08-00689-s001.pdf]

Supplementary Materials: Drinking Water Intake Is Associated with Higher Diet Quality among French Adults

Rozenn Gazan, Juliette Sondey, Matthieu Maillot, Isabelle Guelinckx and Anne Lluch

Table S1. Food categorisations [1].

Food Groups	Subgroups
Fruits and vegetables	Vegetables (cooked and raw) and soup
	Fresh and processed fruits
	Dried fruits and oilseeds
Starches	Refined starches
	Not refined starches
	Ready to eat cereals
Meats/Fishes/Eggs	Eggs
	Fishes
	Meats
Mixed dishes and sandwiches	Mixed dishes
	Sandwiches and savoury puff pastries
Dairy products	Milk
	Fresh dairies
	Cheese
Sweet products	Dairy dessert
	Cake, pies, sweet pastries
	Biscuits and sweets
Drinking water	Tap water
	Non-carbonated bottled water
	Carbonated bottled water
Beverages	Hot drinks (tea and coffee)
	Diet sweet beverages
	Sweet beverages
	Fruit juices
Added fats	Animal fats
	Vegetable fats
	Spices and sauces

[1] Subgroups considered as a fluid are in bold.

Table S2. Fat intakes (% energy) and micronutrient intakes (mg/day or µg/day) across tertiles by gender (means are adjusted for energy) [1].

	Men							Women						
		Tertile of Drinking Water Intake							Tertile of Drinking Water Intake					
	All	≤474 mL/Day	474–879 mL/Day	>879 mL/Day				All	≤500 mL/Day	500–934 mL/Day	>934 mL/Day			
**Variables**	**Mean ± SD**	**Mean ± SD**	**Mean ± SD**	**Mean ± SD**	***p*** **†**	***p*** **‡**	***p*** **for Trend**	**Mean ± SD**	**Mean ± SD**	**Mean ± SD**	**Mean ± SD**	***p*** **†**	***p*** **‡**	***p*** **for Trend §**
Linoleic acid (%energy)	4.9 ± 2.0	4.8 ± 2.0	4.9 ± 2.0	4.9 ± 2.1	0.813	0.569		5.3 ± 1.8	5.2 ± 1.9	5.3 ± 1.6	5.3 ± 2.0	0.800	0.904	
α-Linolenic acid (%energy)	0.5 ± 0.2	0.5 ± 0.3	0.4 ± 0.2	0.5 ± 0.3	0.085	0.053		0.5 ± 0.2	0.5 ± 0.2	0.5 ± 0.2	0.5 ± 0.2	0.017	0.017	0.877
Magnesium (mg/day)	344.2 ± 5.2	328.9 ± 4.1	340.7 ± 5.1	363.1 ± 6.2	<0001	0.016	0.004	287.5 ± 4.3	265.5 ± 3.7	289.6 ± 3.9	307.4 ± 5.4	<0001	<0001	<0001
Calcium (mg/day)	971.0 ± 20.5	944.5 ± 26.0	948.2 ± 18.4	1020.3 ± 17.2	0.006	0.026	0.053	836.4 ± 15.3	752.8 ± 14.0	837.6 ± 14.7	918.9 ± 17.2	<0001	<0001	<0001
Copper (mg/day)	1.7 ± 0.1	1.6 ± 0.1	1.6 ± 0.1	1.7 ± 0.1	0.310	0.126		1.4 ± 0.1	1.3 ± 0.1	1.4 ± 0.1	1.6 ± 0.1	0.005	0.004	0.001
Iron (mg/day)	13.0 ± 0.2	12.9 ± 0.2	13.3 ± 0.2	13.0 ± 0.2	0.392	0.566		10.5 ± 0.2	9.9 ± 0.1	10.4 ± 0.2	11.1 ± 0.2	<0001	<0001	<0001
Phosphorus (mg/day)	1411.0 ± 14.9	1423.8 ± 19.7	1409.7 ± 12.7	1399.6 ± 12.4	0.564	0.218		1114.0 ± 12.3	1089.5 ± 12.0	1121.0 ± 12.1	1131.6 ± 12.9	0.055	0.149	
Iodin (mg/day)	142.8 ± 3.3	142.6 ± 3.6	143.2 ± 3.2	142.6 ± 3.2	0.986	0.958		121.2 ± 2.3	115.4 ± 2.2	121.5 ± 2.0	126.7 ± 2.6	0.003	0.038	0.011
Selenium (mg/day)	86.5 ± 1.9	87.4 ± 2.4	87.9 ± 2.2	84.2 ± 1.2	0.211	0.232		77.7 ± 2.0	75.4 ± 1.7	79.2 ± 2.4	78.5 ± 1.8	0.141	0.334	
Zinc (mg/day)	12.1 ± 0.2	12.3 ± 0.2	12.3 ± 0.2	11.9 ± 0.2	0.098	0.085		9.0 ± 0.1	8.7 ± 0.1	9.1 ± 0.2	9.2 ± 0.1	0.024	0.013	0.005
Vitamin A (µg/day)	1250.1 ± 55.9	1239.2 ± 72.0	1203.3 ± 49.3	1307.7 ± 46.3	0.206	0.020 *	0.161	1071.4 ± 43.9	1032.2 ± 49.1	1044.2 ± 32.7	1137.9 ± 49.8	0.145	0.128	
Vitamin B_1 (mg/day)	1.3 ± 0.1	1.3 ± 0.1	1.3 ± 0.1	1.3 ± 0.1	0.891	0.606		1.1 ± 0.1	1.1 ± 0.1	1.1 ± 0.1	1.2 ± 0.1	0.033 *	0.270	
Vitamin B_2 (mg/day)	1.8 ± 0.1	1.9 ± 0.1	1.8 ± 0.1	1.8 ± 0.1	0.524	0.218		1.6 ± 0.1	1.5 ± 0.1	1.6 ± 0.1	1.6 ± 0.1	0.030 *	0.222	
Niacin (mg/day)	21.6 ± 0.4	21.2 ± 0.4	21.6 ± 0.5	22.2 ± 0.3	0.134	0.497		16.7 ± 0.4	15.8 ± 0.3	16.6 ± 0.3	17.6 ± 0.5	0.007 *	0.084	
Pantothenic acid (mg/day)	5.6 ± 0.1	5.8 ± 0.1	5.6 ± 0.1	5.6 ± 0.1	0.365	0.094		4.6 ± 0.1	4.5 ± 0.1	4.6 ± 0.1	4.8 ± 0.1	0.014 *	0.078	
Vitamin B_6 (mg/day)	1.9± 0.1	1.9 ± 0.1	1.9 ± 0.1	1.9 ± 0.1	0.978	0.811		1.6 ± 0.1	1.5 ± 0.1	1.5 ± 0.1	1.6 ± 0.1	0.002	0.032	0.009
Folic Acid (mg/day)	307.5 ± 6.0	300.1 ± 6.7	307.9 ± 6.0	314.6 ± 5.4	0.251	0.557		271.6 ± 5.6	257.0 ± 5.5	271.0 ± 5.6	286.8 ± 5.6	0.001 *	0.009 *	0.002
Vitamin B_{12} (µg/day)	6.6 ±0.3	6.8 ± 0.3	6.7 ± 0.3	6.5 ± 0.3	0.769	0.751		5.2 ± 0.2	4.9 ± 0.2	5.1 ± 0.2	5.4 ± 0.2	0.164	0.199	
Vitamin C (mg/day)	91.9 ± 3.8	90.1 ± 3.6	89.4 ± 3.6	96.4 ± 4.3	0.482	0.326		95.2 ± 3.1	85.6 ± 2.7	100.4 ± 3.7	99.7 ± 3.0	0.001	0.002	0.002
Vitamin D (µg/day))	2.8 ± 0.1	2.7 ± 0.1	2.8 ± 0.1	3.0 ± 0.1	0.088	0.068		2.5 ± 0.1	2.6 ± 0.2	2.5 ± 0.1	2.5 ± 0.1	0.838	0.740	
Vitamin E (mg/day)	13.2 ± 0.5	12.4 ± 0.5	13.3 ± 0.5	13.9 ± 0.4	0.065	0.150		12.3 ± 0.3	11.9 ± 0.3	12.1 ± 0.3	12.8 ± 0.3	0.080	0.247	

[1] Values are survey-weighted means with standard deviations; † adjustment for energy intake (except for energy and variables expressed in % energy); ‡ adjustment for energy intake (except for energy and variables expressed in % energy), level of education, socio-occupational group, season, level of physical activity, smoker status, region, and quintile of income per consumption unit (ICU); § calculated only for significant differences.
